# Preclinical activity of sacituzumab govitecan (IMMU-132) in uterine and ovarian carcinosarcomas

**DOI:** 10.18632/oncotarget.27342

**Published:** 2020-02-04

**Authors:** Salvatore Lopez, Emanuele Perrone, Stefania Bellone, Elena Bonazzoli, Burak Zeybek, Chanhee Han, Joan Tymon-Rosario, Gary Altwerger, Gulden Menderes, Anna Bianchi, Luca Zammataro, Aranzazu Manzano, Paola Manara, Elena Ratner, Dan-Arin Silasi, Gloria S. Huang, Masoud Azodi, Peter E. Schwartz, Francesco Raspagliesi, Roberto Angioli, Natalia Buza, Pei Hui, Heather M. Bond, Alessandro D. Santin

**Affiliations:** ^1^Department of Obstetrics, Gynecology and Reproductive Sciences, Yale University School of Medicine, New Haven, CT, USA; ^2^Department of Experimental and Clinical Medicine, Magna Graecia University, Catanzaro, Italy; ^3^Department of Gynecologic Oncology, IRCCS National Cancer Institute, Milan, Italy; ^4^University Campus Bio Medico of Rome, Department of Obstetrics and Gynecology, Rome, Italy; ^5^Department of Pathology, Yale University School of Medicine, New Haven, CT, USA; ^6^Department of Clinical and Experimental Medicine, Laboratory of Molecular Haematopoiesis and Stem Cell Biology, University “Magna Græcia”, Catanzaro, Italy

**Keywords:** sacituzumab govitecan, IMMU-132, uterine carcinosarcoma, trop-2

## Abstract

**Background:**

Uterine and ovarian carcinosarcomas (CS) are rare cancers with poor prognosis. Sacituzumab-govitecan (SG) is a new class of antibody-drug-conjugate (ADC) targeting the human-trophoblast-cell-surface marker (Trop-2) conjugated with the active metabolite of irinotecan (SN-38). We evaluated the efficacy of SG against biologically aggressive CS.

**Methods:**

Trop-2 expression was evaluated in 10 formalin-fixed-paraffined-embedded (FFPE) CS by immunohistochemistry and 9 primary CS cell-lines by flow-cytometry. One Trop-2 low/negative (SARARK14) and two Trop-2 positive (SARARK4, SARARK9) cell-lines were tested in cell-viability assays . The *in vivo* antitumor activity of SG was tested in xenografts models (ie, SARARK9) with strong Trop-2 expression.

**Results:**

Strong/diffuse staining was seen in 30% (3/10) of FFPE tumors and 33% (3/9) of primary CS cell lines. Trop-2 positive cell-lines (SARARK4, SARARK9) showed higher sensitivity to SG *in vitro* when compared to Trop-2 low/negative (SARARK14) cell lines. In xenografts, twice-weekly intravenous administration of SG for three weeks showed a significant tumor growth inhibition when compared to control, to ADC control and to the naked AB (p=0.004, p=0.007 and p=0.0007, respectively). SG significantly improved overall survival at 90 days when compared to control groups (p<0.0001).

**Conclusion:**

SG may represent a novel class of active drugs for carcinosarcomas patients overexpressing Trop-2.

## INTRODUCTION

Carcinosarcomas (CS), also known as mixed malignant Mullerian tumors (MMMT), account for less than 5% of all gynecologic cancers [1]. Carcinosarcomas can arise from the ovary, cervix or uterus and are composed of an epithelial component as well as a sarcomatous component. The sarcomatous component can be classified as either homologous or heterologous based on the presence of tissues that are either native or foreign to the uterus respectively. Many theories regarding the mechanism of development of this tumor, composed of two dissimilar cell populations, have been proposed but the latest evidence suggest that CS develops as a result of epithelial to mesenchymal transformation [2, 3]. Standard treatment for CS is aggressive surgical debulking followed by chemotherapy with or without radiation. Despite aggressive surgical and adjuvant therapy, 5-year survival rates remain poor [4]. New therapies are needed to improve CS patient outcomes. Recently, whole exome studies have reported the genetic landscape of a large number of CS, identifying new deranged pathways as potential therapeutic targets [2] [5] [6]. Consistent with these results, novel targeted therapies in ovarian and uterine carcinosarcomas have been proposed [7].

Antibody-drug conjugates (ADCs) represent a new class of drugs that combine a surface receptor targeting antibody with a cytotoxic small molecule allowing selective delivery of a chemotherapeutic agent to tumor cells. The ADC strategy not only enhances the therapeutic window of cytotoxic drugs but also minimizes chemo-related side effects. For example, trastuzumab emtansine (TDM-1) showed promising results in HER2 amplified breast cancer [8] and it was approved by the Food and Drug Administration (FDA) in 2013 [9]. Furthermore, the chemical structure of the linkers (non-cleavable vs cleavable) provide peculiar characteristics to the different ADCs. Non-cleavable linkers manifest anti-tumor activity only after internalization while ADCs with cleavable linkers can kill not only the antigen-positive target cells but also the surrounding antigen-negative cells and they therefore be more suitable for treatment of tumors with heterogeneous antigen expression.

Trop-2, a 45kDa transmembrane glycoprotein encoded by the gene *TACSTD2* of chromosome 1p32, is a cell surface glycoprotein which was originally identified in human placenta trophoblastic tissue and that possesses the ability to invade uterine decidua during placental implantation [10]. Although the biological role of Trop-2 is still unclear, its overexpression has been found to be related to invasiveness and poor prognosis in multiple human carcinomas [11–15]. Notably, Trop-2 is highly expressed on the surface of many epithelial tumors when compared to normal cells, and this feature makes Trop-2 an excellent target for ADCs [16–19]. Trop-2 overexpression among uterine cancers has been previously reported as high as 96% in endometrioid endometrial cancers and 65% in uterine serous carcinoma (USC) [20, 21]. Sacituzumab govitecan (SG) is a new class of ADC targeting Trop-2 antigen to deliver SN-38, the active metabolite of irinotecan, which has a 100- to 1,000 fold higher potency than irinotecan. In contrast to other ADCs SG has a hydrolysable linker (CL2A) supporting a time released bystander effect in the tumor environment, SN-38 causes single-stranded DNA breaks that progress into double-stranded breaks if unrepaired leading to activation of the intrinsic apoptotic pathway and cell death [16, 22–24]. Recently, there have been multiple clinical trials in a variety of advanced solid cancers including breast, urothelial cancer, small cell lung cancer and non-small cell lung cancer that have shown encouraging therapeutic activity of SG [18, 25–28]. The objective of this study was to evaluate the expression of Trop-2 in CS tissues and primary CS cell lines and to examine the preclinical anti-tumor activity of SG *in vitro* and *in vivo* against multiple primary CS models and xenografts. We demonstrate for the first time that SG is highly active, both *in vitro* as well as *in vivo*. Clinical studies with SG in patients harboring Trop-2 overexpressing CS resistant to chemotherapy are warranted.

## RESULTS

### Trop-2 expression in CS patient samples by Tissue Microarray (TMA) and in primary CS cell lines by flow cytometry

**Figure 1:**
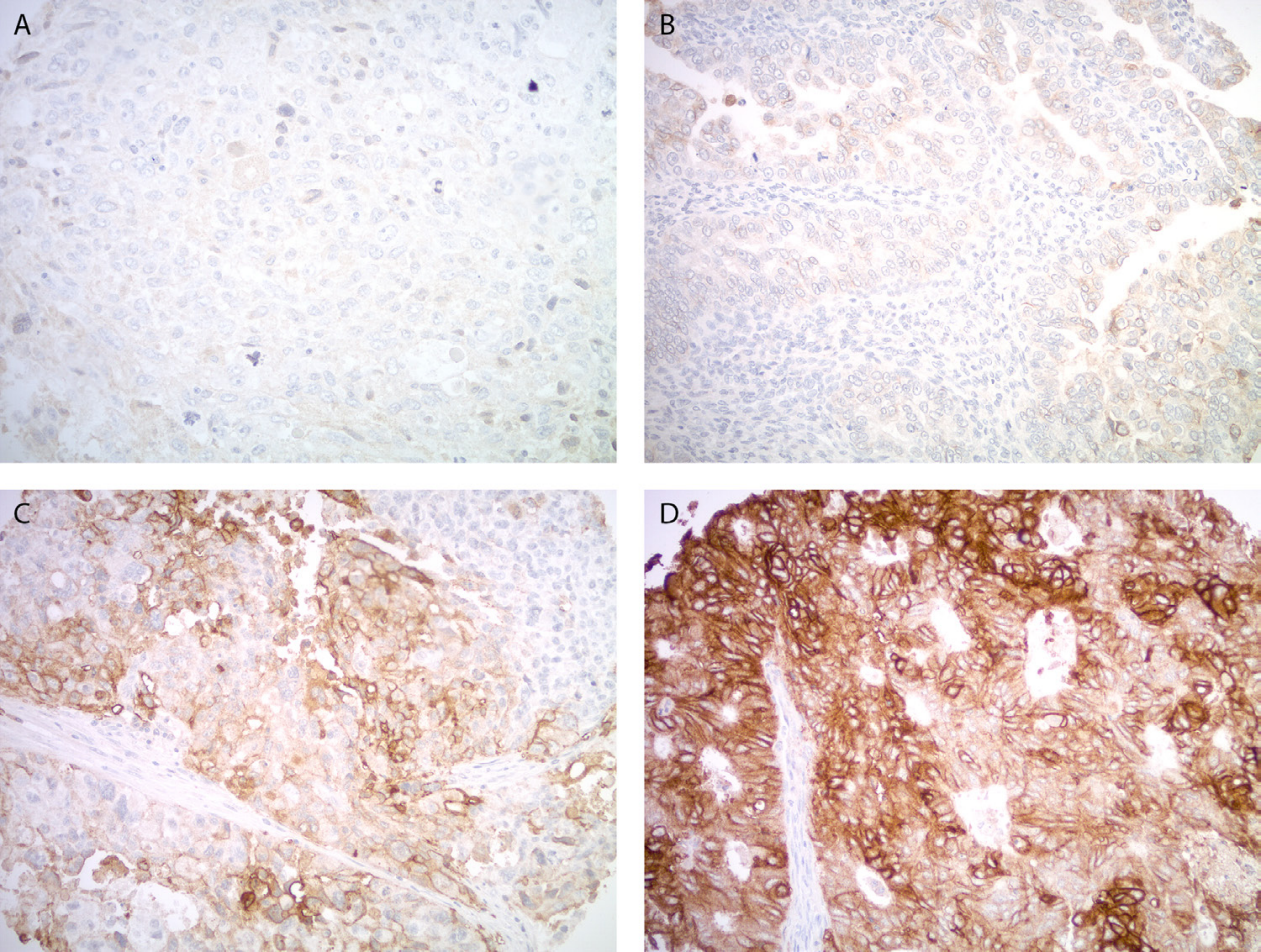
Trop-2 expression by immunohistochemistry in uterine carcinosarcoma. Representative images from the tissue microarray show no Trop-2 immunostaining **(A)**, weak focal **(B)**, moderate focal **(C)** and strong diffuse **(D)** Trop-2 expression in the carcinoma components. All images at 200x original magnification.

**Figure 2:**
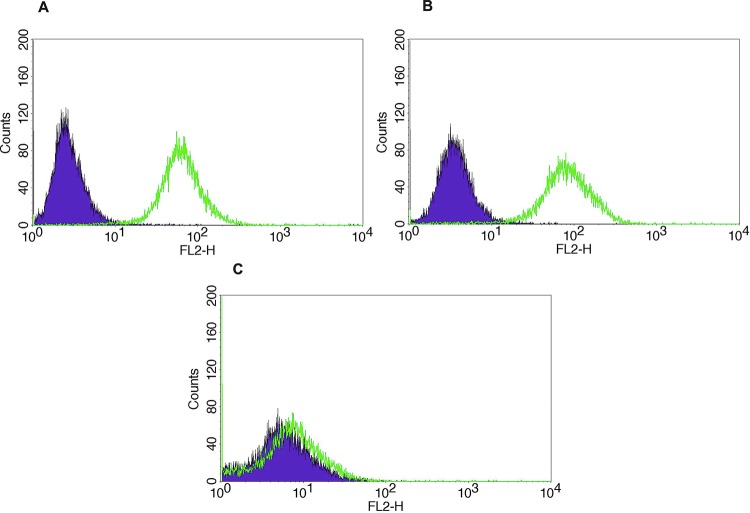
Trop-2 flow cytometry results of representative primary CS cell lines. **(A)** SARARK4 (Trop-2), **(B)** SARARK9 (Trop-2), **(C)** SARARK14 (Trop-2-).

A TMA was used to semi-quantitatively analyze Trop-2 expression by IHC. Out of 10 primary CS, strong diffuse (score 3) staining was seen in 30% (3/10). Representative IHC images are presented in Figure 1. Nine primary CS cell lines were established as described in Methods. Tumor characteristics including stage, histology and primary site are shown in supplemental data (Supplementary Table 1). Three out of nine (33%) of the CS cell lines were determined to have strong (2+) Trop-2 expression by flow cytometry. A representative flow cytometry histogram of three representative primary CS cell lines showing 2+ (SARARK4, SARARK9) and 0 (SARARK14) Trop-2 expression, is shown in Figure 2.

### *In vitro* viability assays

**Figure 3:**
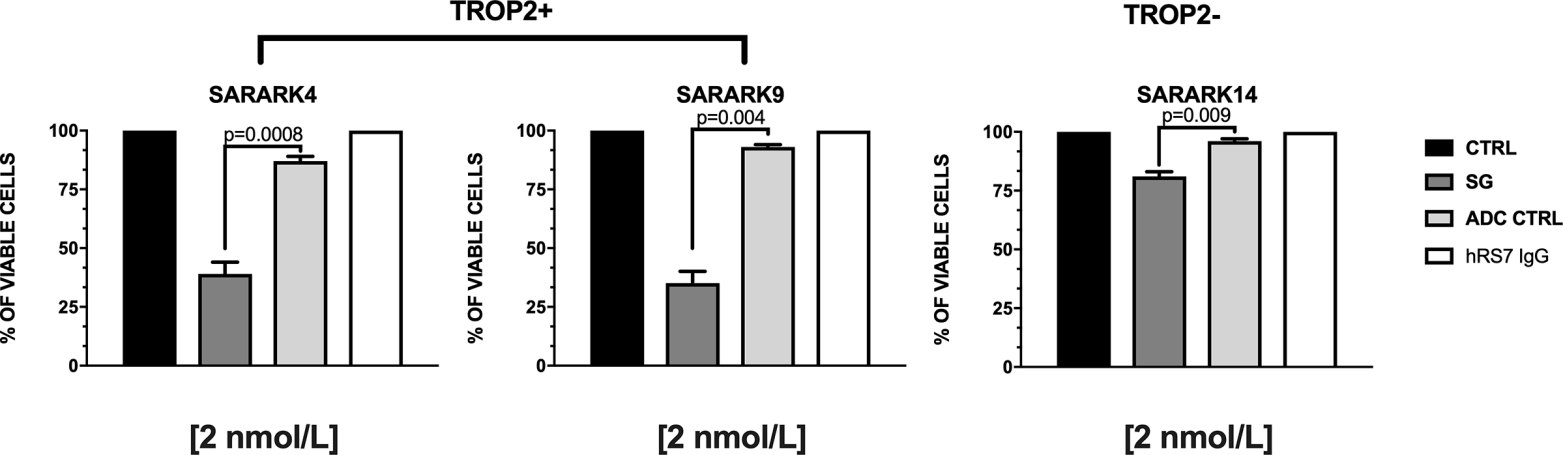
Cell viability assay. Three primary CS cell lines (ie, SARARK4 and SARARK9, Trop-2 positive and SARARK14, Trop-2 negative) were used. Cell viability was determined as described in methods. SG demonstrated significantly more potent cytotoxicity when compared to the ADC isotype control in Trop-2 positive cell lines. No cell killing was observed with hRS7 IgG (naked AB) in any of cell lines in the absence of effecter cells (ie, NK cells).

Three primary CS cell lines with similar *in vitro* growth (ie, SARARK4, SARARK9, Trop-2 positive and SARARK14, Trop-2 low/negative) (Supplementary Table 1) were used for *in vitro* viability assays. Cell viability was determined as described in methods. As shown in Figure 3, SG demonstrated significantly more potent cytotoxicity when compared to the ADC isotype control in Trop-2 positive cell lines (SARARK4 and SARARK9, p=0.0008 and p=0.004 respectively) (Figure 3 and Supplementary Table 1). Although SG induced a statistically significant cytotoxicity when compared to the ADC isotype control in Trop-2 negative cell line (i.e., low Trop-2 expression), SG demonstrated significantly more potent cytotoxicity in Trop-2 positive cell lines (SARARK4 and SARARK9) when compared to the Trop-2 low/negative cell line (SARARK14) (p=0.001 and p=0.002, respectively). No cell killing was observed against any of the cell line tested after challenged with naked AB in the absence of effector cells (ie, NK cells).

### Bystander effect *in vitro*

**Figure 4:**
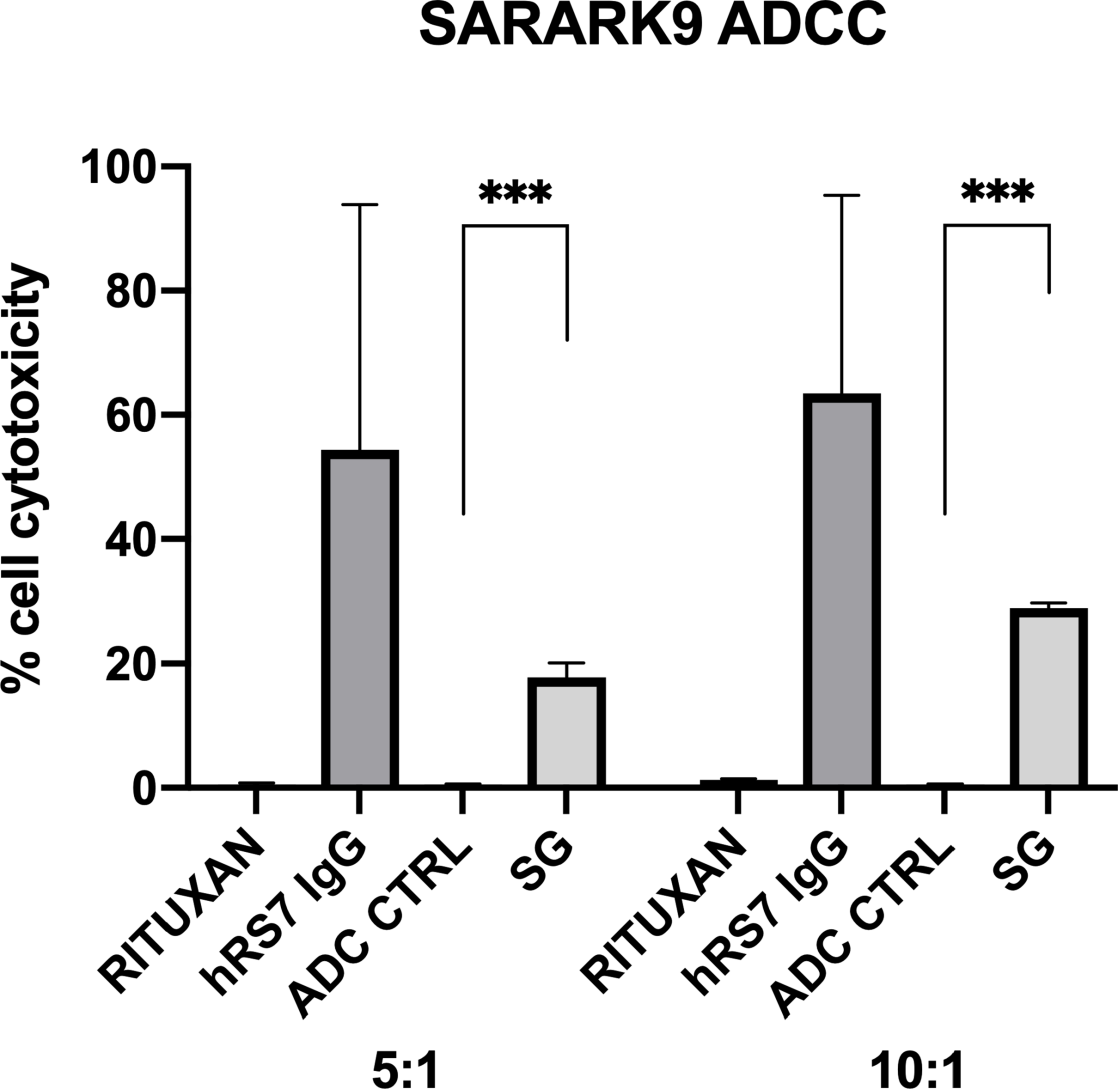
Bystander effect assay. Bystander killing effect was evaluated by admixing SARARK9 (i.e., high Trop-2 expression) *in vitro* with low/negligible Trop-2 expressing cells (i.e., GFP-ARK4 cells). A significant increase in cytotoxicity of ARK4 cells was seen when ARK4 and SARARK9 were cultured together and treated with SG when compared to ADC-control-treated ARK4 co-cocultures (p=0.017).

To evaluate the ability of SG to induce a bystander killing effect in a tumor environment where Trop-2 is expressed heterogeneously we tested the ADC activity by admixing SARARK9 (ie, high Trop-2 expression) *in vitro* with low/negligible Trop-2 expressing cells (i.e., GFP-ARK4 cells) for 72 hrs (cells were incubated with the drugs for 12 hrs as stated in the materials and methods section). As shown in Figure 4, a significant increase in cytotoxicity of ARK4 cells was seen when ARK4 and SARARK9 were cultured together and treated with SG when compared to ADC-control-treated co-cultures (p=0.017).

### SG and hRS7 IgG induce ADCC against Trop-2-positive primary CS

**Figure 5:**
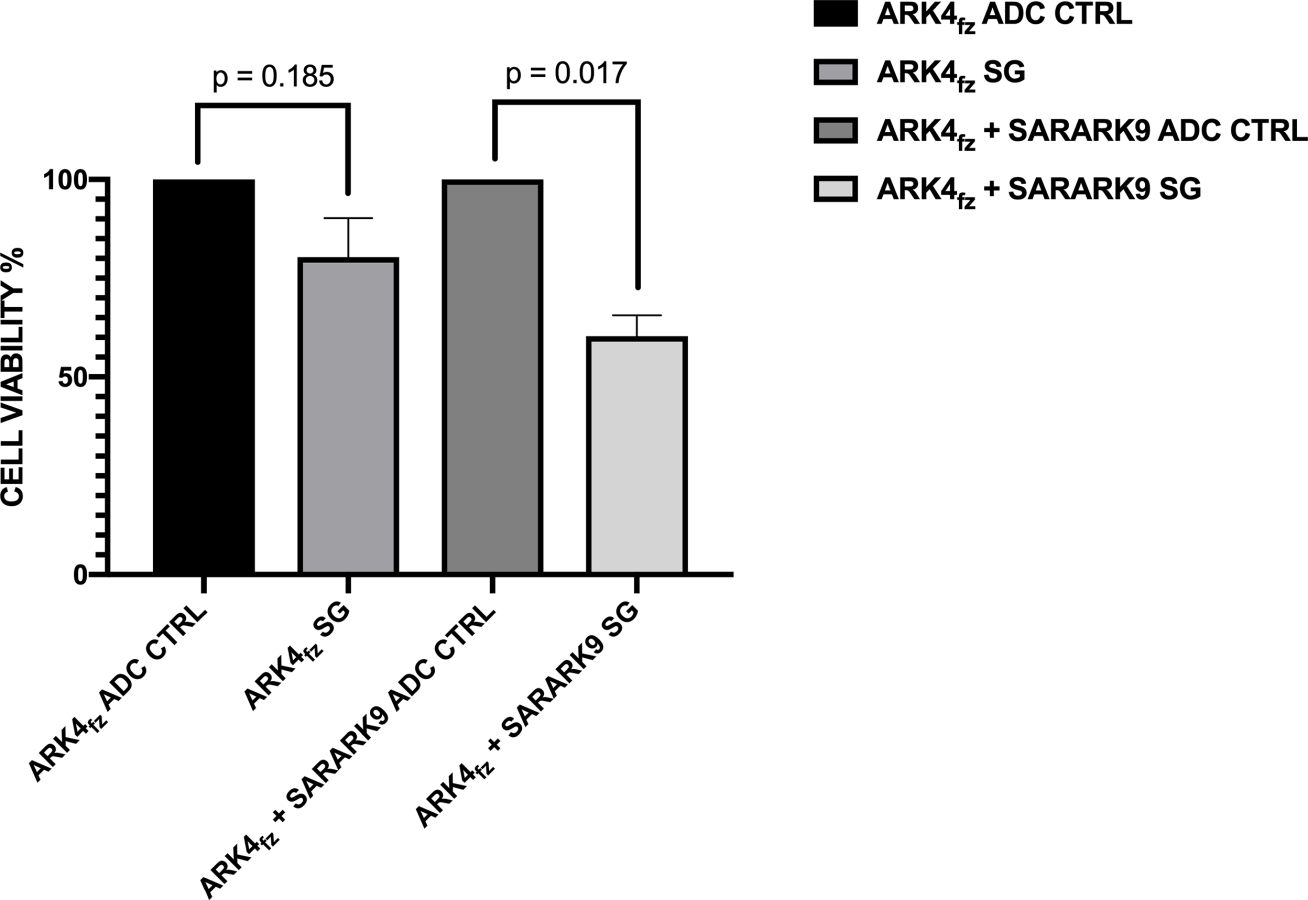
Antibody-dependent cellular cytotoxicity. A representative primary CS cell line (SARARK9, 2+ Trop-2 positive) was tested for ADCC. No ADCC effect was detected when combined with PBLs and isotype control antibody (Rituximab). Experiments were then performed in the presence of SG, ADC control, and hRS7-IgG (naked AB). Trop-2 positive cell line (SARARK9) induced ADCC at high levels in the presence of both SG and hRS7-IgG. In contrast, no significant ADCC was seen in presence of ADC control (*** indicate p<0.05)

A representative primary CS cell line (SARARK9, 2+ Trop-2 positive) was tested for ADCC as described in methods. SARARK9 cell line was consistently found to be resistant to PBL-mediated cytotoxicity when combined with PBLs and isotype control antibody (Rituximab) (2 μg/mL) at E:T ratios of 5:1 and 10:1 (mean ± SEM cytotoxicity of 0.56 ± 0.14% and 1.27 ± 0.08%) (Figure 5). Sensitivity experiments were then performed in the presence of SG, ADC control, and naked antibody at 2μg/mL (Figure 5). Trop-2 positive cell line (SARARK9) induced ADCC at high levels in the presence of SG and hRS7-IgG (mean ± SEM cytotoxicity of 17.79 ± 1.34% and 28.95 ± 0.50% for SG and 54.36 ± 22.82% and 63.48 ± 18.39 % for hRS7-IgG, p < 0.05 at ratios of 5:1 and 10:1, respectively). In contrast, no significant ADCC was seen in presence of ADC control mean ± SEM cytotoxicity of 0.50 ± 0.05% and 0.53 ± 0.03% at ratios of 5:1 and 10:1, respectively) (Figure 5).

### *In vivo* antitumor activity

**Figure 6:**
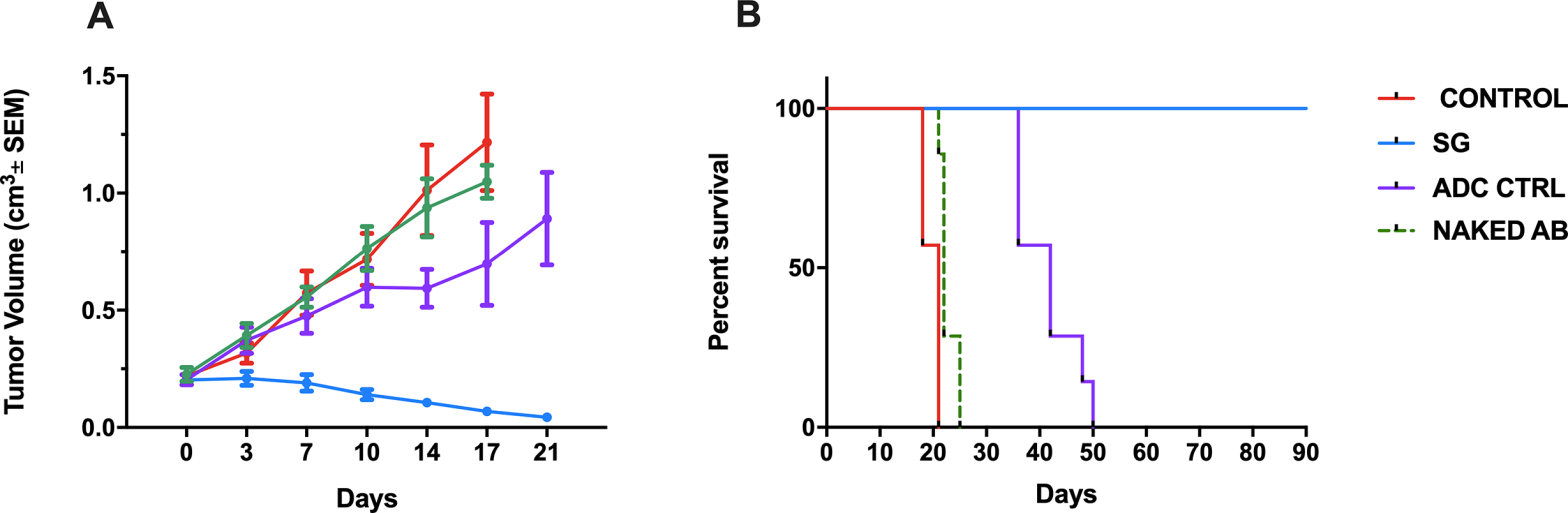
Antitumor activity **(A)** and Survival **(B)** of the SG versus controls in CS xenografts. Mice were treated twice per week for three weeks by IV injection of saline, SG, ADC control, and naked AB as described in Methods. A) SG significantly inhibited tumor growth when compared to saline, ADC control, and naked AB beginning at day 7 of the treatment (p=0.004, p=0.007 and p=0.0007 respectively). B) Overall survival at 90 days was significantly improved in the SG group (p<0.0001).

For *in vivo* experiments we compared the antitumor activity of SG, ADC control, naked AB and saline against Trop-2 positive CS xenografts (SARARK9). SG significantly induced tumor regression when compared to saline, ADC control, and naked AB beginning at day 7 of the treatment with mean tumor volumes of 0.19±0.03cm^3^, 0.57±0.09 cm^3^, 0.47±0.07 cm^3^ and 0.55±0.04 cm^3^, respectively (p=0.004, p=0.007 and p=0.0007 respectively). Of note, three mice treated with SG experienced complete tumor regression. Furthermore, no significant differences in tumor growth inhibition were observed when comparing saline and ADC control or saline and naked AB. This protective effect was evident for the entire treatment period as well as during the 90-day follow-up period, while mice treated with saline, ADC control and naked AB all demonstrated rapid tumor growth (Figure 6A). Overall survival at 90 days was significantly improved in the SG group (p<0.0001) (Figure 6B). Importantly, SG treatment was well tolerated by all the animals with no significant change in body weight (Supplementary Figure 1).

## DISCUSSION

Cancer cells may differentially overexpress specific surface proteins when compared to normal cells which may be used as effective targets for precision medicine tools such as mAb and ADC. Consistent with this view, the epidermal growth factor type II receptor HER2 represents the cornerstone in the treatment of HER2 overexpressing breast cancers [29] and it has been recently demonstrated effective as therapeutic target against USC [30].

Trop-2 was first described forty years ago, however, its expression, role, and function became of great interest only few years later, after the discovery of Trop-2 overexpression on the surface of many different types of epithelial tumors [31].

Moreover, it has been shown that Trop-2 is able to promote cell proliferation by inhibiting apoptosis and accelerating cell cycle progression through increased expression of antiapoptotic genes such as BCL-2 [31]. CS are rare but highly aggressive gynecologic cancers composed of both an epithelial and sarcomatous component. The rarity, aggressive biologic nature, and rapid development of chemotherapeutic resistance make these tumors difficult to cure and extremely difficult to study within clinical trials. Development of new therapies remains an unmet medical need.

In this study we evaluated Trop-2 expression in FFPE CS tumors and primary CS cell lines. We found that up to 33% of CS overexpress Trop-2. Next, we evaluated the preclinical activity of SG against primary CS cell lines and xenograft models. Our study shows, for the first time, SG to be highly effective in Trop-2 positive CS cell lines and in a preclinical model of CS xenograft. Of interest, both SG and naked AB demonstrated significant ADCC against Trop-2 positive cell lines *in vitro*. These results obtained with SG confirmed the results of a previous study from our group in which Trop-2 expressing ovarian cancer cell lines were found highly susceptible to ADCC when exposed to the naked humanized anti-Trop-2 monoclonal antibody hRS7 [32]. Taken together these data suggest that tumor cell killing by SG may not only be limited to the internalization of the ADC and the consequent intracellular release by the hydrolysable linker CL2A of the SN-38 toxic load but is also potentially mediated by immune system cells (ie, NK cells). Furthermore we experimentally demonstrated that SG induced bystander killing of Trop-2 negative tumor cells when admixed with Trop-2 overexpressing tumor cells. This unique feature, due to the hydrolysable nature of the SG linker, may allow delivery of SN-38 not just to targeted cells but also to surrounding cells (ie, Trop-2 negative tumor cells, stromal cell and endothelial cells), leading to an improved therapeutic index, especially in biologically aggressive CS in which two components are present (i.e. epithelial and sarcomatous).

Finally, the preclinical *in vivo* experiments further confirmed the *in vitro* data showing that few injections of SG are highly effective in inducing regression of biologically aggressive CS xenografts. Of interest, SG treatment *in vivo* induced tumor growth inhibition in all treated animals with complete tumor regression in three out of seven mice. Importantly, treatment was well tolerated, with no significant weight change documented in animals for the entire study.

The efficacy and safety of SG have been recently demonstrated in clinical trials targeting multiple solid tumors, but no study as yet reported SG activity against gynecologic cancer patients [18, 25–28]. In the first human trial in subjects with various types of metastatic epithelial cancers, 24% of patients (6/25) achieved long-term survival (> 15 months), 8% of patients (2/25) had a partial response (PR), and 64% of patients (16/25) had stable disease (SD) as their best response [18]. Fatlas et al reported the result of SG in metastatic platinum-resistant urothelial cancers [25]. SG showed a clinically significant response in 50% of patients (3/6), with progression-free survival of 6.7 to 8.2 months and overall survival of 7.5+ to 11.4+ months. SG was also reported to be active against heavily pretreated metastatic lung cancers, both non-small cell lung cancers (mNSCLC) and small cell lung cancers (mSCLC). The clinical benefit rate (complete response (CR) + PR +SD ≥ 4 months) was 43% for mNSCLC and 39% for mSCLC [26, 27]. Recently, encouraging results with SG were reported in heavily pretreated metastatic triple-negative breast cancers (mTNBC)[28]. 108 patients with mTNBC received a median of 3 previous therapies (range, 2 to 10). The response rate (3 complete and 33 partial responses) was 33.3% (95% confidence interval [CI], 24.6 to 43.1), and the median duration of response was 7.7 months (95% CI, 4.9 to 10.8). The clinical benefit rate was 45.4%. Median progression-free survival was 5.5 months (95% CI, 4.1 to 6.3), and overall survival was 13.0 months (95% CI, 11.2 to 13.7). Myelotoxic effects were the main adverse reactions [28]. Grade ≥ 3 neutropenia was the most common side effect (30% to 42%) reported in clinical trials, but neutropenic fever occurred only in 2% to 9% of patients [26–28].

In conclusion, our results demonstrate that 1) Trop-2 is overexpressed in ~ 33% of CS, 2) primary CS cell lines overexpressing Trop-2 are highly susceptible to killing *in vitro* by SG, 3) SG in the presence of effector cells (NK cells) may induce significant ADCC against Trop-2 positive CS cells, 4) SG demonstrated a significant bystander killing effect, which could aid in treating tumors with heterogeneous antigen expression such as CS and 5) SG is highly effective in Trop-2+ CS xenografts. These preclinical results combined with recent phase II data demonstrating significant clinical responses in multiple solid tumors resistant to chemotherapy, strongly support the design of clinical trials in Trop-2 positive recurrent CS patients.

## MATERIALS AND METHODS

### Establishment of CS cell lines and Trop-2 expression analysis

This study was approved by the institutional review board and was carried out according to the declaration of Helsinki. Consents were obtained from all patients per institutional guidelines prior to tissue collection. The CS cell lines were established from fresh tumor biopsy samples as described previously [33-35]. Briefly, solid tumors were minced finely in an enzymatic solution of 0.14% collagenase type I (Sigma-Aldrich, St. Louis, MO) and 0.01% DNAse (Sigma-Aldrich, St. Louis, MO) in RPMI 1640 (GibcoLife *Technologies, Carlsbad*, *CA*). Enzymatically dissociated tumors were then washed twice in RPMI 1640 with 10% fetal bovine serum (FBS, Gemini, Calabasas, CA) and plated in Petri dishes using RPMI 1640, 10% FBS, 1% penicillin with streptomycin (Mediatech, Manassas, VA), and 1% amphotericin (Life Technologies, Carlsbad, CA). The cell lines were kept in an incubator at 37°C with 5% CO_2_ and continually monitored for growth. The experiments were performed with primary cell lines with limited passages (<30). Tumors were staged per the International Federation of Gynecology and Obstetrics staging system.

### Tissue microarray

A retrospective CS cohort represented in TMA format was used in this study (CS N = 10). In brief, representative areas from primary tumors were selected in hematoxylin/eosin–stained sections. Formalin-fixed paraffin-embedded FFPE TMAs were constructed to include 0.6 mm tissue cores from CS with duplicate cores for each case. Tissue sections were cut at 5 μm and purified goat polyclonal antibody against the recombinant human Trop-2 extracellular domain (R&D Systems, Inc., Minneapolis, MN; diluted 1:100) was applied for 1 hr. A secondary biotinylated anti-goat antibody (Vector Laboratories, Burlingame, CA; diluted 1:250) and the streptavidin–biotin complex (StreptABComplex/HRP, Dako, CA) were applied, then 303-diaminobenzidine (Dako) was used as chromogen and the sections were counterstained by hematoxylin (Dako). Appropriate negative and positive controls were used. The percentage of tumor cells with membranous Trop-2 immunoreactivity was estimated and the staining intensity was assessed semi-quantitatively as follows: 0, no staining; 1+, weak; 2+, moderate and 3+, strong staining. The final immunoreactivity score was calculated by multiplying the staining intensity (1+, 2+, 3+) with the percentage of positive tumor cells, and was classified in 4 ordinal categories: 0-9 negative (score 0), 10-99 weak (score 1), 100-199 moderate (score 2), and 200-300 strong (score 3) (Figures 1, 2). Specific consents or waivers under an approved Yale Human Investigation committee protocol were obtained prior to processing any tissues.

### Determination of Trop2 expression in CS cell lines

CS cell lines were analyzed by flow cytometry for Trop-2 expression after being cultured *in vitro* for up to 30 passages. Briefly, the CS cell lines were incubated with 2.5 μg/mL of naked AB (hRS7 IgG) for 30 minutes at room temperature. For staining, a fluorescein isothiocyanate-conjugated goat anti-human F(ab1)2 immunoglobulin (FITC) was used as a secondary reagent (BioSource International). Analysis was conducted with a FACScalibur, using Cell Quest software (BD Biosciences, San Diego, CA). Data analysis for mean fluorescence intensity (MFI) was performed using Cell Quest (BD Biosciences) and Prism 7.01. Cell lines with an MFI greater than 50 were determined to have 2+ expression of Trop-2, while cell lines with an MFI of 20 to 50 were noted to have 1+ and 20 or less was 0.

### Drug

SG, non-targeting ADC control (h679-CL2A-SN-38), and naked AB were obtained from Immunomedics, Inc. (Morris Plains, NJ). SG and ADC control were dissolved in sterile 0.9% sodium chloride as a 10μM stock solution for the *in vitro* experiment. The drug-to-antibody ratio (DAR) of SG was 6.78, and that of ADC control was 6.84. For the *in vitro* experiment, the dosage of the drug was adjusted according to the DAR, in order that equivalent quantities of SN-38 were used in SG and ADC control treated cells. For the *in vivo* experiment, SG and ADC control were dissolved in sterile 0.9% sodium chloride as a 5mg/mL solution. The drug amount was not adjusted for the *in vivo* experiments since SN-38 does not possess significant toxicity; therefore, small differences would not affect the *in vivo* results. hRS7 IgG (molecular weight: 150kDa) was obtained in liquid form from Immunomedics, Inc as a 10mg/mL solution.

### *In vitro* cell viability assay

Since, as previously described [17], the majority of SN-38 associated with SG is released into the culture media during the 4-day incubation period, and therefore ADC potency in *in vitro* experiments would be similar to that of SN-38 alone, a different strategy was required to demonstrate SG *in vitro* activity. Briefly, three CS cell lines (SARARK4, SARARK9, SARARK14) with similar doubling time were selected and were incubated at 37°C in 15 ml falcon tubes at a density of 20,000 to 40,000 cells for 12 hrs with 2 nM of SG, ADC control and hRS7 IgG. The cells were then washed to remove unbound conjugate and plated in 6-well tissue culture plates in RPMI 1640 media (Life Technologies) supplemented with 10% fetal bovine serum, 1% penicillin/streptomycin (Mediatech), and 0.3% fungizone (Life Technologies). Four days after drug treatment, cells were harvested in their entirety, centrifuged and stained with propidium iodide (2 µL of 500 mg/mL stock solution in PBS). Analysis was performed using a flow cytometry–based assay to quantify percent viable cells as a mean±SEM relative to untreated cells as 100% viable controls. A minimum of three independent experiments was performed.

### Bystander effect

Briefly, a 1:1 ratio of 2+ Trop-2 expressing CS (i.e., SARARK9) and TROP-2 negative uterine serous carcinoma cells (i.e., ARK4) stably transfected with a green fluorescence protein (GFP) plasmid (pCDH-CMV-MCSEF1-copGFP, a gift from Dr. Simona Colla, MDACC), were mixed (40,000 cells/well of each cell type) and plated in 6-well plates (2 mL/well). After an overnight incubation, cells were treated with SG or isotype ADC control for 12 hrs. The cells were then washed to remove unbound conjugate and, after additional 72 hrs, cells were collected, centrifuged and stained with propidium iodide (2µl of 500 µg/mL stock solution in PBS) to identify percentages of live/dead cells in each well. Flow cytometry based assay was performed to quantify live cells as a mean ± SEM relative to untreated cells. Bystander effect was then assessed by comparing the percentage of Trop-2 negative (ARK4) live cells when they were co-cultured with Trop-2 positive cells (SARARK9) versus Trop-2 negative live cells that were incubated alone.

### Tests for antibody-dependent cellular cytotoxicity (ADCC)

Standard 4-h chromium (^51^Cr) release assays were performed to measure the cytotoxic reactivity of Ficoll–Paque-PLUS (GE Healthcare, Kings Park, NY, USA) separated peripheral blood lymphocytes (PBLs) from several healthy donors against primary CS cell lines at effector to target ratio (E:T) of 5:1 and 10:1. The release of ^51^Cr from target cells was measured as evidence of tumor cell lysis after exposure of the tumor cells to a concentration of 2.5 µg/ml of SG, ADC control, or hRS7 IgG. Tumor cells incubated with PBLs without an ADC were used as negative controls. Chimeric anti-CD20 mAb rituximab (2.5 μg/ml) was used in all bioassays as a negative control for hRS7. As a positive control condition, 1% sodium dodecyl sulfate (SDS) was used to achieve complete lysis of target cells. A gamma radiation counter (2470 WIZARD2 Automatic Gamma Counter, PerkinElmer) was used to count the ^51^Cr released from the target cells. ADCC of SG, ADC control, or hRS7 IgG was calculated by the following formula: % cytotoxicity = 100 × (E − S)/(T − S), where E is the experimental release, S is the spontaneous release by target cells, and T is the maximum release by target cells lysed with 0.1% SDS. Results are mean ± SEM.

### *In vivo* testing

A representative cell line, SARARK9 (Trop-2+), was injected into 5-8 week old severe combined immunodeficiency (SCID) mice subcutaneously (ENVIGO, Indianapolis, IN). Each mouse was injected with 7 million cells suspended in approximately 300 µL of a 1:1 solution of sterile PBS containing cells and Matrigel® (BD Biosciences). After the tumor volume reached 0.2 cm^3^, the mice were randomized into four groups (7 mice/group): control, SG, ADC control, naked AB. SG, ADC control, and hRS7 were given at the dose of 500 μg IV twice per week for three weeks (i.e., day 1, 4, 8, 11, 15, and 18) and then mice were observed for overall survival analysis. Tumor volume and mice weight were recorded twice weekly. Tumor volume was calculated as (length x width^2^)/2. Animal care and euthanasia were carried out according to the rules and regulations as set forth by the Institutional Animal Care and Use Committee (IACUC).

### Statistical analysis

Statistical analysis was performed using Graph Pad Prism 7 (GraphPad Software, Inc. San Diego, CA). The differences in the inhibition of proliferation in the CS cell lines after exposure to treatments were evaluated by two-tailed unpaired student t-test. Tumor volume differences at specific time points were compared using an unpaired t-test. Survival curves were compared using the log-rank test. A *p*-value < 0.05 was considered to be significant.

## SUPPLEMENTARY MATERIALS FIGURES AND TABLES




