# Supplementary material for: Preclinical activity of sacituzumab govitecan (IMMU-132) in uterine and ovarian carcinosarcomas
Source: Oncotarget. 2020 Feb 4;11(5):560–70. doi: 10.18632/oncotarget.27342 (PMC7007291; doi:10.18632/oncotarget.27342)
Supplement: Supplementary file 1 [file oncotarget-11-560-s001.pdf]

Preclinical activity of sacituzumab govitecan (IMMU-132) in uterine and ovarian carcinosarcomas

SUPPLEMENTARY MATERIALS

Supplementary Table 1: Cell lines characteristics and Trop2 expression

CELL LINE	AGE	RACE	FIGO* STAGE	PRIMARY SITE	HISTOLOGY		TROP2 MFI	SCORE
					EC	SC		
**SARARK1**	70	A	IC	Uterus	END-CC	Homologous ESS	20,5	1+
**SARARK3**	74	W	IIIC	Ovary	SER	Heterologous CDRS	437,4	2+
**SARARK4**	77	W	IIIC	Ovary	SER	Heterologous CDRS	201,9	2+
**SARARK7**	55	W	IV	Ovary	CC-SER	Heterologous CDRS	0,5	0
**SARARK9**	66	W	IIIC2+	Uterus	SER	Homologous ESS	271,4	2+
**SARARK11**	67	W	IIIC1	Uterus	END	Heterologous CDRS	15,1	0
**SARARK12**	39	W	IVB	Uterus	SER	Homologous ESS	5,3	0
**SARARK13**	72	W	IVB	Uterus	SER	Heterologous CDRS	7,2	0
**SARARK14**	59	B	IVB	Uterus	SER END	Homologous ESS	5,9	0

Abbreviations: *FIGO, International Federation of Gynecology and Obstetrics; CC, clear cell; CDR, chondroid; CDRS, chondrosarcoma; EC, epithelial component; END, endometrioid; ESS, endometrial stromal sarcoma; MFI, mean fluorescence intensity; SC, sarcomatous component; SER, serous.
