# Supplementary material for: Preclinical activity of sacituzumab govitecan (IMMU-132) in uterine and ovarian carcinosarcomas
Source: Oncotarget. 2020 Feb 4;11(5):560–70. doi: 10.18632/oncotarget.27342 (PMC7007291; doi:10.18632/oncotarget.27342)
Supplement: Supplementary file 2 [file oncotarget-11-560-s002.pdf]

Preclinical activity of sacituzumab govitecan (IMMU-132) in uterine and ovarian carcinosarcomas

SUPPLEMENTARY FIGURE

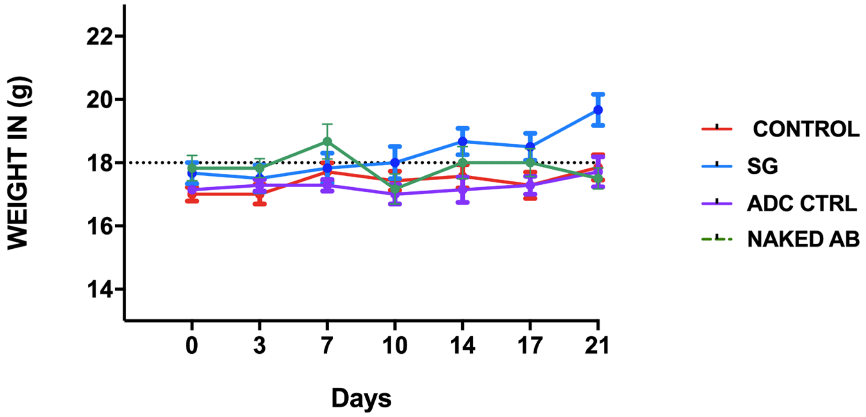

Supplementary Figure 1: Variation in body weight of the mice treated. SG treatment was well tolerated by all the animals with no significant change in body weight (P>0.05)
